# Interactions between FGFR2 and RSK2—implications for breast cancer prognosis

**DOI:** 10.1007/s13277-016-5266-9

**Published:** 2016-07-30

**Authors:** Dominika Czaplinska, Kamil Mieczkowski, Anna Supernat, Andrzej C. Skladanowski, Radzislaw Kordek, Wojciech Biernat, Anna J. Zaczek, Hanna M. Romanska, Rafal Sadej

**Affiliations:** 1 Department of Cell Biology, Intercollegiate Faculty of Biotechnology, University of Gdańsk and Medical University of Gdańsk, 80-210 Gdańsk, Poland; 2 Department of Molecular Enzymology, Intercollegiate Faculty of Biotechnology, University of Gdańsk and Medical University of Gdańsk, 80-210 Gdansk, Poland; 3 Department of Pathology, Medical University of Łódź, 92-213 Łódź, Poland; 4 Department of Pathomorphology, Medical University of Gdańsk, Gdańsk, Poland

**Keywords:** RSK2, FGFR2, Breast cancer

## Abstract

We have previously demonstrated that fibroblast growth factor receptor 2 (FGFR2) activates ribosomal s6 kinase 2 (RSK2) in mammary epithelial cells and that this pathway promotes in vitro cell growth and migration. Potential clinical significance of FGFR2 and RSK2 association has never been investigated. Herein, we have undertaken an evaluation of a possible relationship between FGFR2/RSK2 interdependence and disease outcome in breast cancer (BCa) patients. The clinical analysis was complemented by an in vitro investigation of an involvement of RSK2 in the regulation of FGFR2 function. Primary tumour samples from 152 stage I–III BCa patients were examined for FGFR2 and RSK2 gene and protein expression. FGFR2 showed a positive correlation with RSK2 at both protein (*p* = 0.003) and messenger RNA (mRNA) (*p* = 0.001) levels. Lack of both FGFR2 and activated RSK (RSK-P) significantly correlated with better disease-free survival (DFS) (*p* = 0.01). Patients with tumours displaying immunoreactivity for either or both FGFR2 and RSK-P had 4.89-fold higher risk of recurrence when compared to the FGFR2/RSK-P-negative subgroup. FGFR2-RSK2 interactions were verified by co-immunoprecipitation and internalization assays in HB2 mammary epithelial cell line (characterized by high endogenous FGFR2 and RSK2 expression). In vitro analyses revealed that FGFR2 and RSK2 formed an indirect complex and that activated RSK exerted a significant impact on fibroblast growth factor 2 (FGF2)-triggered internalization of FGFR2. Our results suggest that the FGFR2-RSK2 signalling pathway is involved in pathophysiology of BCa and evaluation of FGFR2/RSK-P expression may be useful in disease prognostication.

**Electronic supplementary material:**

The online version of this article (doi:10.1007/s13277-016-5266-9) contains supplementary material, which is available to authorized users.

## Background

Fibroblast growth factor receptors (FGFRs) and their ligands (FGFs) play an important role in mammalian ontogenesis, angiogenesis and wound healing. FGFR family consists of four tyrosine kinase receptors (FGFR1–4) that are expressed in multiple alternatively spliced variants, which define ligand specificity and downstream signalling pathways [1]. FGFR2 has been implicated in development and progression of breast cancer (BCa). Genome-wide analysis has identified single nucleotide polymorphisms (SNPs) in intron 2 of the *FGFR2* gene associated with a higher risk of BCa [2]. In BCa patients, increased both nuclear and cytoplasmic FGFR2 expression correlated with lower overall and disease-free survival [3]. *FGFR2* gene amplification was reported in approximately 1–2 % of all breast cancers [4] and in about 4 % of triple-negative tumours (TNBC) [5]. Preclinical studies of FGFR-specific small molecule inhibitors provide evidence to suggest that *FGFR2* amplification might serve as a new therapeutic target, especially in TNBC, notoriously resistant to currently available therapies [5]. In accordance with that, a number of experimental studies in various BCa models demonstrated high efficiency of FGFR inhibitors in the induction of tumour growth arrest [6–8]. In addition, FGFR2 was shown to contribute to the maintenance of tumour-initiating cells (TICs), a subpopulation with increased tumourigenic potential, self-renewal, heterogeneous differentiation and bipotency. TICs isolated from human and mice mammary tumours were found to be enriched with FGFR2-overexpressing population [9]. It has also been reported that activation of FGFR2 enhanced invasive growth of human BCa cells in mice [10], thus implicating FGFR2 in both initiation and progression of the disease.

Ribosomal S6 kinase 2 (RSK2) is a member of the serine/threonine kinase family consisting of four isoforms (RSK1–4) in humans. Altered RSK signalling was found to support cell transformation and tumour growth. Overexpression of RSK2 has been associated with several types of hematologic and epithelial malignancies including breast cancer [11, 12]. Expression of RSK2 was elevated in about 50 % of mammary tumours [13]. In BCa patients, *RSK2* gene expression correlated with poor disease-free survival [14]. Additionally, it was demonstrated that specific inhibitors and small interfering RNA (siRNA) targeting RSK2 significantly suppressed growth and ability to self-renewal of TIC population within TNBC and delayed tumour initiation in mice [14]. RSK2 was also found to mediate the pro-migratory functions of ERK/MEK pathway. A genome-wide messenger RNA (mRNA) expression analysis revealed that MEK/ERK➔RSK signalling regulates expression of 53 genes from diverse pathways crucial for mammary cell motility and invasiveness [15].

In canonical pathway, RSK kinases are activated by MAPK/ERK signalling in response to several growth factors, peptide hormones and neurotransmitters, e.g. epidermal growth factor (EGF), insulin and IGF-1 [16–18]. Alternative mechanisms of RSK activation, including those mediated by tyrosine kinase receptors, are being currently investigated. We recently identified a new signalling pathway where fibroblast growth factor 2 (FGF2)/FGFR2 indirectly activated RSK2 at Tyr529 by p38 kinase in normal mammary and BCa cell lines. This pathway was shown to co-exist with the classical MEK/ERK-driven activation of RSK2. In addition, we demonstrated that RSK2 was involved in FGF2/FGFR2-driven formation of focal adhesions, cell migration and anchorage-independent growth of BCa cells [19]. A number of other reports have shown various interrelations between members of FGFR and RSK families. For instance, FGFR3 has been proved to directly phosphorylate RSK2, which is known to play a critical role in haematopoietic transformation [20]. The association between the FGFR2 and RSK1 was shown to be involved in FGFR2-induced AKT activation in epithelial cells [21]. On the other hand, RSK2 appears to directly phosphorylate and regulate endocytosis of FGFR1 in osteosarcoma cells. Interaction between FGFR1 and RSK2 has been demonstrated in yeast two-hybrid system and cell cultures [22]. Existing knowledge of FGFR/RSK interdependence is almost exclusively based on in vitro studies in various cellular models; however, it is becoming increasingly evident that this association might have important functional implications. The main objective of this study was, therefore, to examine a possible clinical significance of FGFR2/RSK2 interdependence at the gene and protein levels in BCa patients, as well as to reveal molecular basis of an involvement of RSK2 in the regulation of FGFR2 function in mammary epithelial cells.

Both clinical material analyses and in vitro experiments confirmed the postulated FGFR2/RSK2 interdependence. In primary tumour samples of BCa, we found a positive, statistically significant correlation between FGFR2 and RSK2 expression at both mRNA and protein levels. Importantly, phosphorylated RSK (RSK-P) as well as combined expression of either or both FGFR2 and RSK-P was associated with poor disease-free survival. RSK2 and FGFR2 were shown to form a transient, indirect complex in mammary epithelial cells in vitro. RSK activity was also identified to regulate FGFR2 internalization in response to ligand (FGF2) binding. Taken together, our results indicate that FGFR2/RSK2 signalling loop may participate in BCa progression and be predictive of poor outcome in patients with breast carcinoma.

## Materials and methods

### Patient selection and samples

The study group included 152 patients with invasive breast cancer (characteristics of the cohort are summarized in Table 1) treated between 1999 and 2009 at the Medical University Hospital in Gdansk. Primary tumour samples were obtained by surgical excision or excisional biopsy prior to any systemic treatment. Median age of the patients was 57 years (range 27–86 years, average 58 years). Follow-up data were available for 147 patients; median follow-up time was 50.5 months (range 1.6–103.5 months). Thirty-two patients (21.05 %) developed tumour recurrence, and 23 (15.13 %) died of BCa. Oestrogen receptor (ER) and progesterone receptor (PgR) were scored according to the classical Allred system with cut-point 3 for the positive result. Human epidermal growth factor receptor 2 (HER2) status was assessed according to the HercepTest criteria (Dako), with 3+ score defining positive result. Scores (2+) were tested by FISH for HER2 gene amplification with the PathVysion® HER-DNA probe kit (Abbott Molecular), as recommended by the manufacturer.

**Table 1:** Patient characteristics

Patient characteristics (*N* = 152)		
Age	27–86 (average 58)	
	*N*	%
T stage		
T1	53	34.87 %
T2	78	51.32 %
T3	9	5.92 %
T4	11	7.24 %
Missing data	1	0.66 %
N stage		
N0	68	44.74 %
N1	50	32.89 %
N2	27	17.76 %
N3	5	3.29 %
Missing data	2	1.32 %
Grade		
G1	9	5.92 %
G2	80	52.63 %
G3	45	29.61 %
Missing data	18	11.84 %
HER2		
Negative	107	70.39 %
Positive	21	13.82 %
Missing data	24	15.79 %
ER		0.00 %
Negative	63	41.45 %
Positive	86	56.58 %
Missing data	3	1.97 %
PgR		
Negative	54	35.53 %
Positive	95	62.50 %
Missing data	3	1.97 %
Histological type		
Ductal	104	68.42 %
Lobular	21	13.82 %
Other	14	9.21 %
Missing data	13	8.55 %
Molecular subtype		
HR+, HER2−	83	54.61 %
HR+, HER2+	9	5.92 %
HR−, HER2+	14	9.21 %
TNBC	21	13.82 %
Missing data	25	16.45 %

### RNA extraction and reverse transcription

After collection, tissue samples were immediately frozen in liquid nitrogen and stored at −80 °C for further analyses. Twenty to thirty milligrams of tissue was homogenized with zircon beads in MagNA Lyser (Roche) for 40 s. Total RNA was isolated by using RNeasy Mini Kit (Qiagen) according to the manufacturer’s protocol, including on-column DNase I treatment. For all samples, RNA concentration and purity was determined by using the Nano-Drop ND-1000 spectrophotometer (Thermo Scientific, USA). Qualitative analysis of RNA was performed by microcapillary electrophoresis by using the Agilent 2100 Bioanalyser (Expert software version B.02.08) with an RNA 6000 Nano Kit (Agilent Technologies). For each sample, 1 μg of RNA was used as a template in reverse transcription (RT) reaction performed with Transcriptor First Strand cDNA Synthesis Kit (Roche) in a 20 μl volume with random hexamer primers, according to the manufacturer’s protocol.

### qPCR and gene expression analysis

Gene expression level was determined by RT-qPCR in a CFX96 thermal cycler (Bio-Rad). Reaction parameters were as follows: 2 min at 50 °C, 10 min at 95 °C, 40 cycles of 15 s at 95 °C followed by 1 min at 60 °C. Forty nanograms of cDNA in 4 μl was added per reaction and mixed with 10 μl of TaqMan Universal PCR Master Mix (Applied Biosystems, Roche), 5 μl of water and 1 μl of specific primer and probe mix. TaqMan Gene Expression Assays were used to measure expression of *FGFR2* (Hs01552926_m1), *RSK2* (Hs00177936_m1) and two reference genes: *GAPDH* (Hs99999905_m1) and *ACTB* (Hs99999903_m1). For each gene tested, a control without RT reaction was included. Samples were analysed in duplicates, and the average Cq value used as a quantitative value. Relative expression values of each gene were calculated by the delta-delta-Cq method normalized to the reference genes and normal female human mammary tissue (540045, Agilent Technologies) as a calibrator with the use of qBasePLUS software (Biogazelle, ver. 2.0). Median value of gene expression was a cut-off value for positivity.

### Immunohistochemistry on tissue microarrays

TMAs were prepared as described before [23]. Serial 5-μm paraffin sections of formalin-fixed blocks were processed for immunohistochemistry for FGFR2 (mouse anti-human; 1:600; Abnova #H00002263-M01), RSK2 (rabbit anti-human; 1:200; Life Span BioSciences, #LS-B7708) and RSK-P (rabbit anti-human; 1:100, Sigma-Aldrich #SAB4503961) by using protocols recommended by the manufacturers. As a negative control for the immunostaining, primary antibodies were replaced by non-immune sera. Scoring of immunostaining (no distinction was made between subcellular distributions) was carried out as follows: (i) 0/negative—no reactivity or only faint reactivity in <10 % of tumour cells, (ii) 1+/negative—faint reactivity in ≥10 % of tumour cells, (iii) 2+/positive—weak to moderate reactivity in ≥10 % of tumour cells and (iv) 3+/positive—strong reactivity in ≥10 % of the tumour cells. Immunohistochemical staining was evaluated and scored independently by two observers (HMR and RK). The agreement on staining intensity was >90 %. Where there was disagreement, intensity was determined by consensus. Final scores were dichotomized scores into (a) ‘negative’ for 0–1 and (b) ‘positive’ for 2–3.

### Statistical analysis

All statistical analyses were performed by using the STATISTICA software, version 10. Continuous variables were compared by the Spearman’s rank order test. Disease-free survival (DFS) was computed by using Kaplan-Meier method and compared by using *F* Cox test. DFS was defined as the time from surgery to an event (local or distant relapse, second malignancy or death, whichever came first) or censoring. Censoring was defined as lost to follow-up or survival without relapse at the end of follow-up. Cox proportional hazard regression analysis was used to identify the independent predictors of DFS. The results were considered statistically significant when *p* value was lower than 0.05.

### Cell lines, antibodies and reagents

HB2 mammary epithelial cells were purchased from ECACC (Sigma-Aldrich). Cells were grown in DMEM with 10 % FBS, 5 μg/ml insulin, 5 μg/ml hydrocortisone and penicillin/streptomycin (100 U/ml and 100 μg/ml, respectively). Media and their supplements were from Sigma-Aldrich. The following reagents were obtained from Santa Cruz Biotechnology: rabbit anti-FGFR1 (sc-121), rabbit anti-FGFR2 (sc-122), goat anti-RSK2 (sc-1430), mouse anti-ubiquitin (sc-8017) and Protein G PLUS Agarose (sc-2002). Mouse antibody against β-actin (A5316) was obtained from Sigma-Aldrich. The remaining antibodies used in this study were from Cell Signalling Technology: rabbit anti-FGFR-Tyr653/654 (#3471) and rabbit anti-RSK2 (#5528). All growth factors were obtained from PeproTech. Heparin sodium salt, PD173074 inhibitor, ExtrAvidin-Peroxidase and reduced L-glutathione were purchased from Sigma-Aldrich. FMK and BI-D1870 were from AxonMedchem. EZ-link Sulfo-NHS-SS-biotin was obtained from Thermo Scientific.

### Western blotting

Cells grown to 80–90 % confluence were lysed with Laemmli buffer (2× concentrated) containing 2 mM phenylmethylsulfonyl fluoride (PMSF), 10 μg/ml aprotinin, 10 μg/ml leupeptin, 5 mM EGTA, 1 mM EDTA, 2 mM Na_4_P_2_O_7_, 5 mM NaF and 5 mM Na_3_VO_4_. Samples with equal amounts of protein per lane (∼20 μg) were loaded, resolved in SDS polyacrylamide gel electrophoresis (SDS-PAGE) and then transferred onto nitrocellulose membrane. The membranes were incubated for 1 h in 5 % skimmed milk and probed with specific antibodies overnight at 4 °C. Secondary antibodies conjugated with HRP (Sigma-Aldrich) and Western Lightning Plus-ECL (PerkinElmer) were used to visualize specific protein bands.

### Immunoprecipitation

Cells were serum starved overnight, and, when required, media were supplemented with an appropriate inhibitor: FMK (10 μM), BI-D1870 (1 μM) or PD173074 (100 nM). Cells were then stimulated with FGF2 (10 ng/ml) + heparin sulphate (50 ng/ml) or EGF (10 ng/ml) for indicated periods of time before lysis in a suitable buffer. We independently applied relatively harsh (1 % Triton X-100) or mild (0.8 % Brij96/0.2 % Triton X-100) detergent for cell lysis. Lysis buffers were supplemented with the following inhibitors: 2 mM PMSF, 10 μg/ml aprotinin, 10 μg/ml leupeptin, 5 mM EGTA, 1 mM EDTA, 2 mM Na_4_P_2_O_7_, 5 mM NaF and 5 mM Na_3_VO_4_. In the next step, cell lysates were incubated overnight at 4 °C with constant rotation with anti-FGFR1 or anti-FGFR2 antibodies coupled to Protein G Agarose (Santa Cruz Biotechnology, sc-2002). Unbound proteins were removed by extensive beads washing with lysis buffer; protein complexes were eluted from the beads with Laemmli buffer. Samples were resolved by SDS-PAGE and analysed by Western blotting. To analyse FGFR2 ubiquitination, FGFR2 was immunoprecipitated as described above; SDS-PAGE resolved samples were probed with anti-ubiquitin antibodies.

### Internalization assay

Serum-starved cells were placed on ice, washed twice in PBS and labelled for 1 h with 500 μg/ml EZ-link Sulfo-NHS-SS-biotin at 4 °C. Biotin excess was quenched by using 100 mM glycine in PBS. Cells were then incubated with the prewarmed serum-free DMEM containing FGF2 (10 ng/ml) + heparin (50 ng/ml) at 37 °C for 60 min to induce internalization. Plates were placed on ice and treated 2 × 20 min with ice-cold stripping solution (500 mM glutathione (GSH), 75 mM NaCl, 1 mM EDTA, 75 mM NaOH, supplemented with 1 % FBS) to remove biotinylation from proteins that were not internalized from the plasma membrane. After washing with PBS, cells were lysed in 1 % Triton X-100 buffer and FGFR2 was immunoprecipitated as described above. Samples were analysed by SDS-PAGE followed by Western blotting with anti-Avidin-HRP and anti-FGFR2 antibodies.

### Immunofluorescence staining

Cells seeded onto glass coverslips were serum-starved overnight and stimulated with FGF2 for 20 min. Specimens were fixed with 2 % paraformaldehyde in PBS and permeabilized with 0.1 % Triton X-100 in PBS for 1 min. After blocking with 3 % BSA in PBS, cells were incubated with specific primary antibodies: anti-RSK2 (1:75) and anti-FGFR2 (1:100) for 1 h. Cells were then stained with AffiniPure DyLight 549-conjugated or DyLight 488-conjugated secondary antibodies (Jackson ImmunoResearch). Distribution of analysed proteins was examined by using a fluorescence microscope ZEISS AxioVert 200.

## Results

### FGFR2 expression correlated with RSK2 at gene and protein level in BCa

**Fig. 1:**
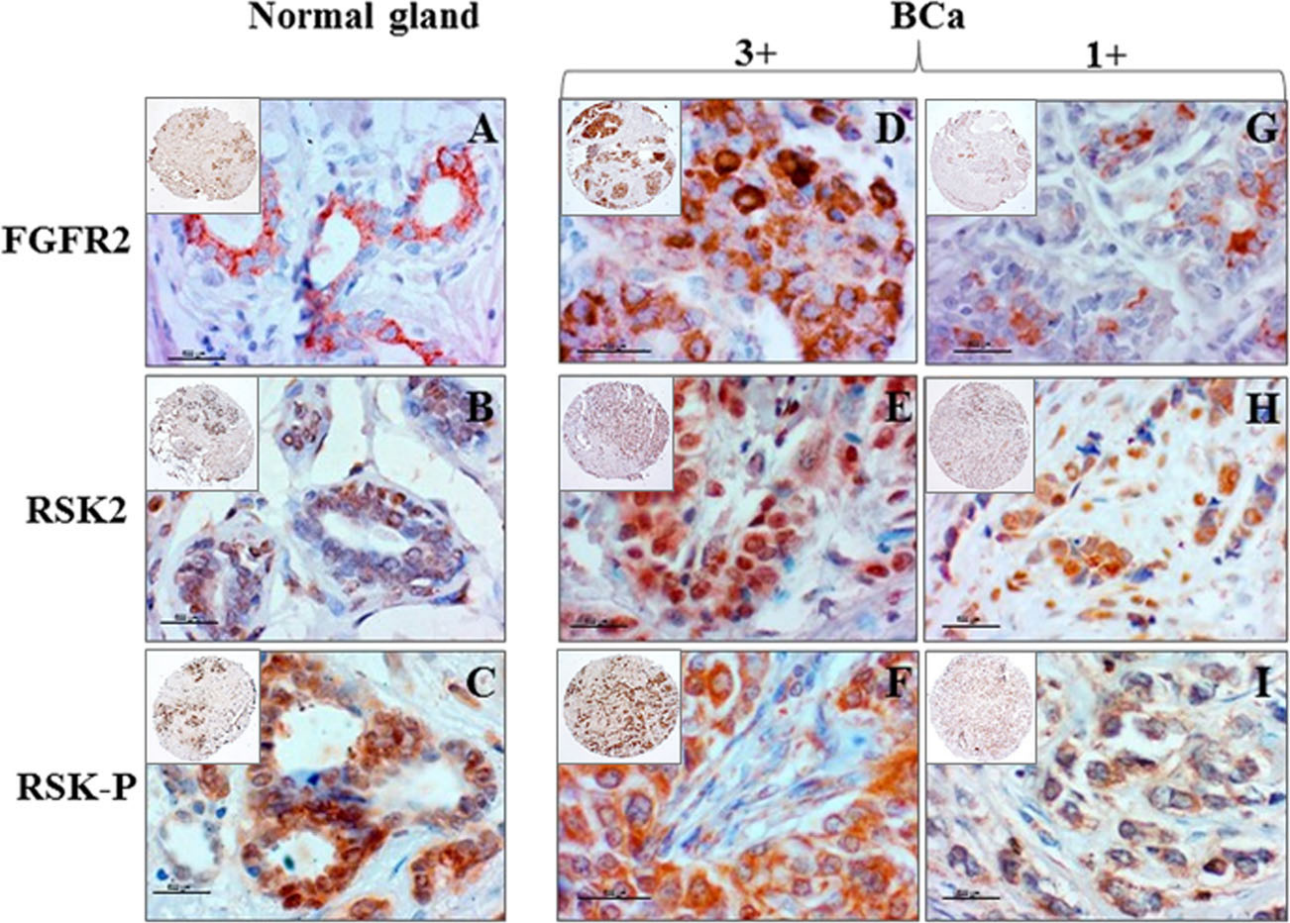
Expression of FGFR2, RSK2 and RSK-P in BCa tissue samples. **a**–**c** In normal gland, the majority of cells displayed moderate/strong expression of **a** cytoplasmic/membranous of FGFR2, **b** cytoplasmic/nuclear RSK2 and **c** cytoplasmic/nuclear RSK-P. Examples of immunoreactivity for FGFR2, RSK and RSK-P in BCa tissue samples scored as 3+ (**d**–**f**) and 1+ (**g**–**i**). Corresponding tissue cores under low magnification (*insets*). *Scale bar* 500 μm

In normal gland, majority of cells displayed moderate/strong expression of (i) cytoplasmic/membranous FGFR2 (Fig. 1a), (ii) cytoplasmic/nuclear RSK2 (Fig. 1b) and (iii) cytoplasmic/nuclear RSK-P (Fig. 1c). In BCa samples, immunoreactivity for all proteins was highly heterogeneous with regards to both cellular localization and level of expression. Examples of levels of expression scored as 3+ (Fig. 1d–f) and 1+ (Fig. 1g–i) are presented on Fig. 1. FGFR2 expression showed a positive, statistically significant association with RSK2 (*p* = 0.003) (Table 2 (a)). Similarly, at the mRNA level, there was a significant correlation between expression of *FGFR2* and *RSK2* genes (*p* = 0.001) (Table 2 (b)). Moreover, values of FGFR2/RSK2 correlation coefficients evaluated in the whole group of patients were increased when analysis was restricted to the TNBC subgroup only (*p* = 0.005 and *p* = 0.015 for gene and protein expression, respectively).

**Table 2:** FGFR2 expression correlates with RSK2 in BCa

	*R* Spearman	*p* value	Number
(A) Samples analysed by IHC for protein expression			
Correlation/protein expression (IHC)			
FGFR2 and RSK2 (whole cohort)	0.267	0.003	121
FGFR2 and RSK2 (TNBC)	0.6	0.015	16
FGFR2 and RSK-P (whole cohort)	0.112	0.6	119
FGFR2 and RSK-P (TNBC)	0.233	0.42	14
(B) Samples analysed by RT-qPCR for gene expression			
Correlation/gene expression (RT-qPCR)			
FGFR2 and RSK2 (whole cohort)	0.32	0.001	98
FGFR2 and RSK2 (TNBC)	0.552	0.005	24

Spearman rank correlation coefficients for FGFR2, RSK2 and RSK-P in the whole cohort and the TNBC subgroup. Numerical values of correlation coefficients, *p* values and number of patients are presented in the corresponding boxes

### FGFR2 and RSK2 status in relation to clinicopathological data

Statistical analysis showed that FGFR2 expression in the whole group of patients correlated only with positive ER status (*p* = 0.0065). Expression of neither RSK2 nor RSK-P was associated with any of clinicopathological variables (Supplementary data, Table 1). At the mRNA level, no significant correlation with clinicopathological features was found for *FGFR2*, while *RSK2* was associated with grade (*p* = 0.02) and PgR (*p* = 0.02) (average gene expression is presented in Supplementary data Table 2).

### Expression of FGFR2 and/or RSK-P is associated with worse DFS

**Fig. 2:**
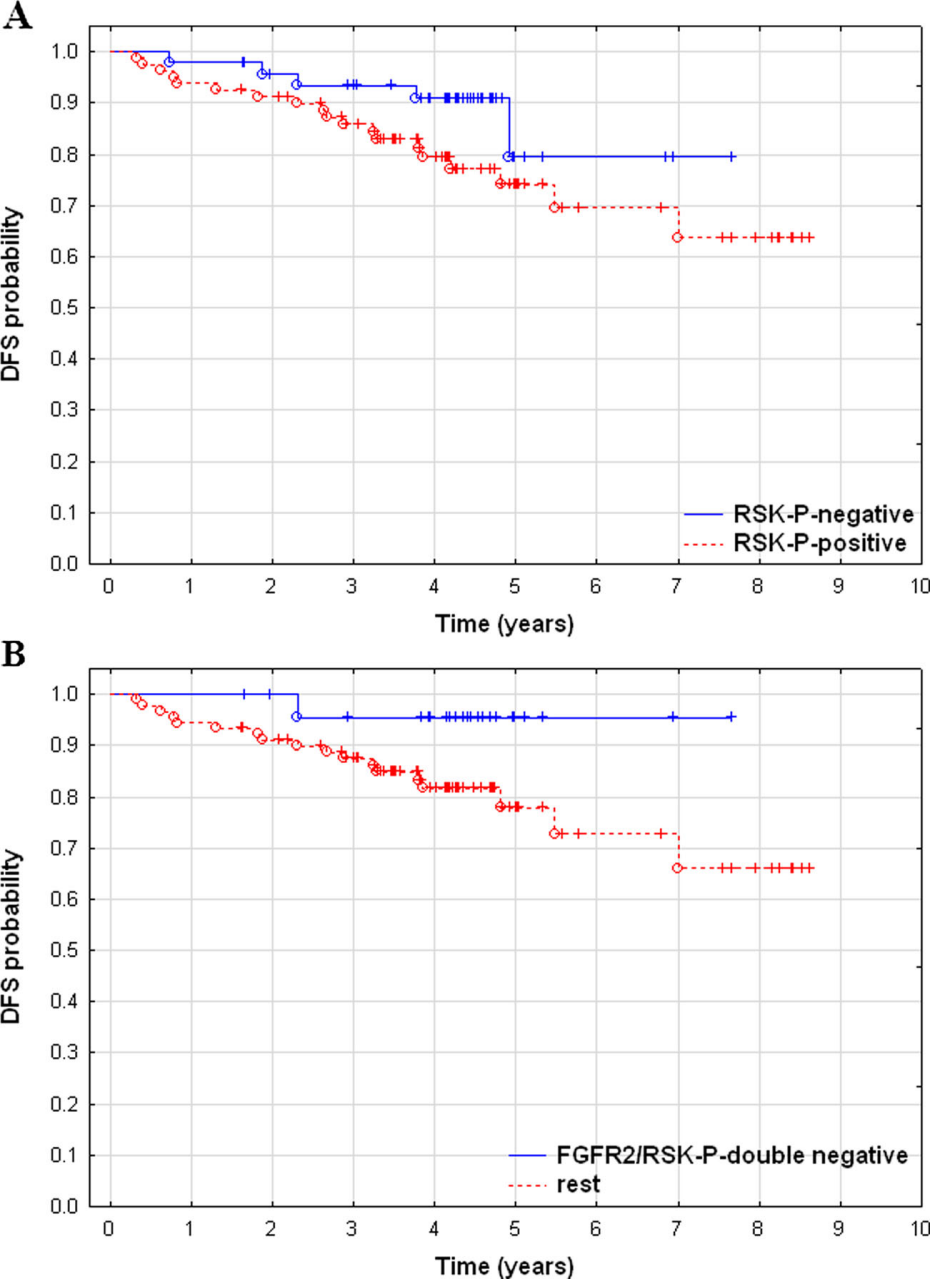
Kaplan-Meier curves according to FGFR2 and RSK-P protein status. **a** Disease-free survival according to RSK-P level (*p* = 0.025). **b** Disease-free survival according to FGFR2/RSK-P level (*p* = 0.01). Patients were divided into the following groups: FGFR2/RSK-P-double negative and rest (positive for either or both FGFR2 and RSK-P)

Patients with tumours positive for RSK-P had 2.134-fold higher risk of recurrence when compared with the RSK-P-negative group. When assessed in combination, expression of either or both RSK-P and FGFR2 was associated with 4.89-fold risk of recurrence compared with the FGFR2/RSK-P-negative patients (Table 3). High expression of RSK-P correlated with worse disease-free survival (*p* = 0.025) (Fig. 2a), whereas lack of both FGFR2 and RSK-P was predictive of better DFS (*p* = 0.01) (Fig. 2b). There was a trend towards statistical significance of RSK-P as an independent marker of recurrence (*p* = 0.065) (Table 3).

**Table 3:** Univariate and multivariate analysis of prognostic factors

	Univariate analysis DFS			Multivariate analysis DFS		
Variable	*N*	Hazard ratio (95 % CI)	*p*	*N*	Hazard ratio (95 % CI)	*p*
Tumour size (T3–4 vs. T1–2)	151	4.745 (2.220–10.142)	0.00006	126	8.409 (3.315–21.334)	0.000007
Lymph nodes (positive vs. negative)	150	2.373 (1.091–5.162)	0.029			NS
ER (positive vs. negative)	149	0.523 (0.257–1.062)	0.073			NS
PgR (positive vs. negative)	149	0.367 (0.180–0.750)	0.006			NS
Histological type (lobular vs. ductal)	135	0.611 (0.184–2.026)	0.421			NS
Grade (G3 vs. G1–2)	132	1.469 (0.652–3.308)	0.353			NS
HER2 status (positive vs. negative)	128	1.348 (0.503–3.612)	0.553			NS
FGFR2 (positive vs. negative)	134	0.973 (0.406–2.3326)	0.950			NS
RSK2 (positive vs. negative)	124	0.699 (0.293–1.666)	0.419			NS
RSK-P (positive vs. negative)	127	2.134 (0.790–5.765)	0.135	126	2.577 (0.943–7.052)	0.065
FGFR2/RSK-P (rest vs. double-negative)	114	4.890 (0.651–36.731)	0.123			NS

### RSK2 and FGFR2 form transient complex in breast cancer cells

**Fig. 3:**
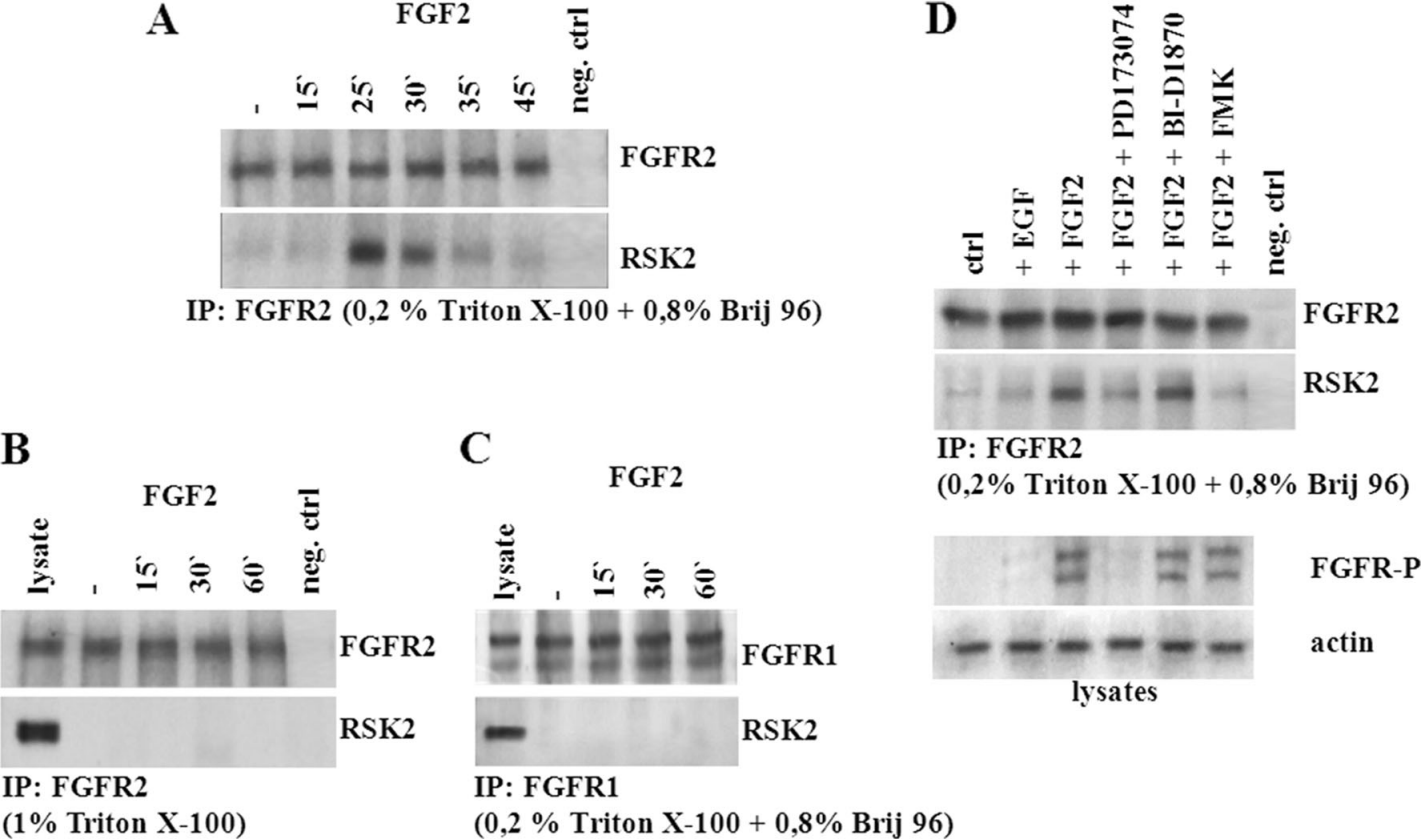
FGFR2 transiently interacts with RSK2 in vitro. Serum-starved mammary epithelial HB2 cells were treated with FGF2 for indicated periods of time. Cell lysates were subjected to co-immunoprecipitation with FGFR2 antibodies in **a** mild (0.8 % Brij96/0.2 % Triton X-100) or **b** harsh (1 % Triton X-100) lysis conditions. **c** Co-immunoprecipitation of FGFR1 and RSK2 by using mild detergent for cell lysis. **d** FGFR2/RSK2 interaction depends on both FGFR and RSK activities. Cells were pretreated with appropriate FGFR or RSK inhibitors in serum-free media and stimulated with FGF2. FGFR2/RSK2 complexes were immunoprecipitated in mild lysis conditions

Interactions between RSK2 and members of the FGFR family have already been reported. For example, phosphorylated RSK2 has been shown to bind to the C-terminal part of FGFR1 participate in regulation of its endocytosis and ubiquitination [22]. In another study, RSK2 was found to be activated in the FGF2/FGFR2-dependent signalling [19]. Herein, we found that FGF2 treatment of HB2 mammary epithelial cell line (characterized by high endogenous FGFR2 and RSK2 expression [19]) triggered co-localization of FGFR2 and RSK2 mostly at the plasma membrane and perinuclear area (Supplementary data. Fig 1). To verify a possible direct FGFR2-RSK2 interaction, we performed co-immunoprecipitation experiments. HB2 cells were serum-starved and treated with FGF2 for indicated periods of time. As shown in Fig. 3a, RSK2 transiently (after 25–30 min of exposure to FGF2) interacted with FGFR2. FGFR2/RSK2 co-precipitation was also confirmed in triple-negative MDA-MB-231 breast cancer cell line (Supplementary data. Fig. 2). Analysed interaction took place only when mild lysis conditions were applied (0.8 % Brij 96/0.2 % Triton X-100). In the presence of a relatively harsh detergent (1 % Triton X-100), the association of RSK2 with FGFR2 was abolished (Fig. 3b). This suggests that RSK2 is a member of larger protein complex interacting with FGFR2 rather than a direct FGFR2 partner. In contrary to the previously published data [22], we did not notice any FGFR1-RSK2 interactions (Fig. 3c).

To further study the nature of the FGFR2-RSK2 cross talk, we evaluated its possible dependence on RSK2 and/or FGFR2 activity. Cells were serum-starved in the presence of appropriate selective inhibitors and stimulated with FGF2 or EGF (used as a control) for 30 min, where indicated (Fig. 3d). Interaction between FGFR2 and RSK2 was observed following stimulation with FGF2, as shown before (Fig. 3a). Interestingly, we noticed little increase in the amount of FGFR2/RSK2 complex following EGF stimulation. Pretreatment with PD173074 (FGFR inhibitor) strongly abolished FGF2-triggered FGFR phosphorylation and FGFR2 association with RSK2. On the other hand, application of BI-D1870, which inhibits RSK-related activation of downstream substrates, leaving the kinase itself phosphorylated, did not affect binding of RSK2 to the FGFR2. In contrary, pretreatment of cells with FMK, which binds in the C-terminal kinase domain of RSK and inhibits RSK autophosphorylation, effectively prevented formation of the RSK2-FGFR2 complex. Efficiency of the inhibitors was tested by using antibodies against RSK-P (Supplementary data. Fig. 3). These results suggest that in mammary epithelial cells, RSK2 forms transient indirect complex with FGFR2 upon FGF2-initiated signalling and that this association depends on activation of both FGFR2 and RSK2, rather than on downstream activities of RSK2.

### RSK activity regulates internalization of FGFR2

**Fig. 4:**
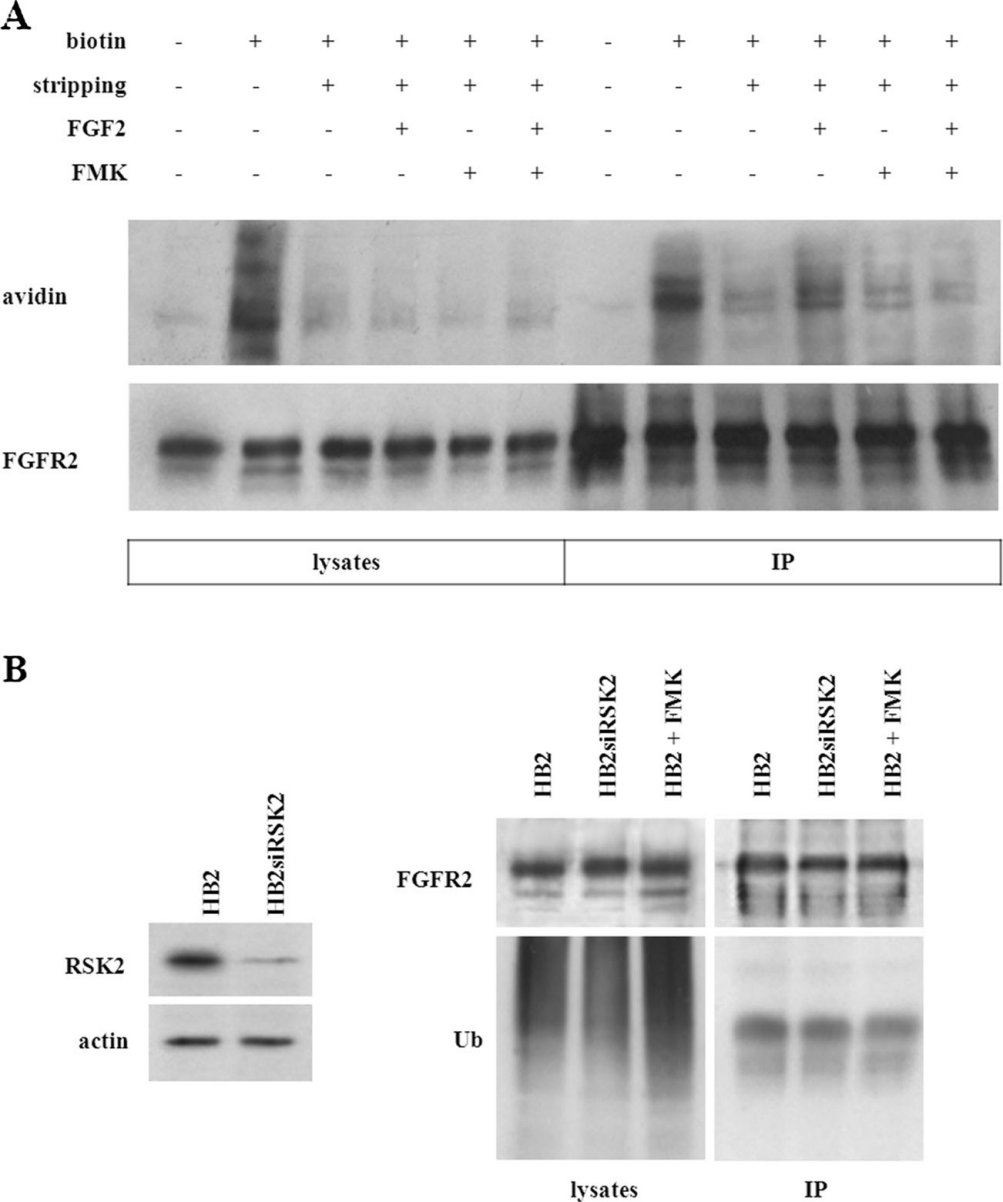
RSK regulates internalization but not turnover of FGFR2. **a** Serum-starved HB2 mammary epithelial cells treated with FMK and/or FGF2 were surface-biotinylated and lysed. FGFR2 was immunoprecipitated and probed for ExtrAvidin. **b** Analysis of FGFR2 ubiquitination in response to RSK2 silencing

Several observations suggest that FGF receptors may undergo endocytosis and regulated trafficking from the cell surface [22, 24, 25]. Here, an involvement of RSK in FGF2-triggered FGFR2 internalization was studied (Fig. 4a). Cells were surface-biotinylated at 0 °C and then stimulated with FGF2. Biotinylated FGFR2 underwent internalization and, therefore, was protected from glutathione stripping. Control samples were surface-biotinylated and immediately washed with GSH buffer to efficiently strip all biotin groups from cell surface proteins (Fig. 4a). Immunoprecipitated FGFR2 was analysed for the level of biotinylation. It has been observed that stimulation with FGF2 promoted FGFR2 internalization. Cell pretreatment with specific RSK inhibitor (FMK) prevented FGF2-mediated FGFR2 internalization suggesting that intracellular trafficking of this receptor is regulated by RSK. We also analysed potential role of RSK in FGFR2 turnover/degradation. RSK2 was transiently knock downed (Fig. 4b), and cells were studied for the level of FGFR2 ubiquitination. We did not find any differences in the amount of expressed FGFR2 or its ubiquitination triggered by RSK2 silencing or inhibition of RSK activity, which suggests that RSK2 takes part in internalization of FGFR2 rather than its degradation.

## Discussion

Expression levels of FGFR2 and RSK2 assessed independently have been reported in several studies as negative prognostic factors for patients with breast carcinoma [2, 3, 14]. As a follow-on from our previous study demonstrating that FGFR2 activates RSK2 in BCa cells in vitro [19], herein we have undertaken an evaluation of a potential clinical significance of an interaction between FGFR2 and RSK2 in breast cancer.

We examined co-expression of FGFR2 and RSK2 in BCa primary tumour samples. In the whole cohort, FGFR2 showed a positive, statistically significant correlation with expression of RSK2 at both protein (*p* = 0.003, Table 2 (a)) and mRNA levels (*p* = 0.001, Table 2 (b)). Interestingly, statistical representation of *FGFR2*/*RSK2* co-expression (correlations coefficients) reached the highest value in the TNBC subgroup (Table 2 (b)), indicating that FGFR2/RSK2 interdependence might be associated with aggressive BCa subtype. Indeed, several studies have already suggested that FGFR2 and RSK2 play an important role in TNBC. siRNA-based screening aimed to identify therapeutic targets for individual breast cancer subtypes recognized *RSK2* as one of three genes with potential clinical significance for the TNBC subgroup [26]. Silencing of RSK2 in triple-negative BCa cells delayed tumour initiation in mice and strongly affected cell survival in vitro [14]. On the other hand, expression of FGFR2 was shown to correlate with decreased overall and disease-free survival of BCa patients [3], and it is well documented that *FGFR2* gene amplification occurs most frequently in the TNBC subgroup. Furthermore, small molecule inhibitors of FGFR turned out to be a promising therapeutic strategy for patients with TNBC, known to poorly respond to currently available treatments [5].

Results of our study showed that RSK-P positivity was associated with shorter DFS of BCa patients. Expression of FGFR2 assessed alone had no prognostic value (data not shown), but analysis of combined immunoreactivity for FGFR2 and RSK-P revealed that lack of both was significantly associated with better DFS (Fig. 2b). In addition, patients with tumours positive for either or both FGFR2 and RSK-P had 4.89-fold higher risk of recurrence when compared to the FGFR2/RSK-P-negative subgroup. Lack of availability of specific antibodies recognizing activated form of FGFR2 or activated RSK2 imposes certain limitations on functional interpretation of these findings. However, in light of our previous report demonstrating an involvement of RSK2 in FGFR2 downstream signalling [19], an assumption that an interaction between FGFR2 and RSK2 promotes BCa progression seems to be fully justified. In contrast to the study by Sun et al. where cytoplasmic expression of FGFR2 was reported to correlate with tumour size [3], our results demonstrated an association of FGFR2 only with ER (*p* = 0.0065) (Supplementary data. Table 1). Discrepancies between these findings may be due to differences in analysed cohorts. While our group comprised various histological BCa subtypes, the study by Sun et al. was restricted only to patients with invasive ductal carcinoma grade 2 (*N* = 125). Moreover, unlike Sun’s study, where nuclear and cytoplasmic expressions of FGFR2 were separately evaluated, differences in FGFR2 subcellular localization were not taken into account in our analysis.

Associations between members of FGFR and RSK families have been previously reported by us and others [19–22]. The present study undertakes an investigation of an FGFR2/RSK2 interdependence in HB2 mammary epithelial cell line characterized by high endogenous FGFR2 and RSK2 expression [19]. Co-immunoprecipitation in serum-starved cells treated with FGF2 revealed a transient interaction between FGFR2 and RSK2 kinase (Fig. 3a). Formation of this complex seemed to be dependent on lysis conditions and occurred only in the presence of a mild detergent (0.8 % Brij 96/0.2 % Triton X-100), which suggests that RSK2 might be a member of a larger FGFR2-interacting protein complex rather than a direct FGFR2 partner. In the control experiment, in contrary to observations reported by Nadratowska-Wesolowska [22], we observed no interaction between FGFR1 and RSK2 (Fig. 3c). This could be due to phenotypic differences between analysed cell lines, especially the FGFR expression profiles, and/or dissimilarities of in vitro stimulation conditions with the FGFR ligands.

Having demonstrated that FGFR2 and RSK2 formed a transient and indirect complex in HB2 cells, we went on to establish whether their association was dependent on RSK2 and/or FGFR2 activity (Fig. 3d). Data presented in the Fig. 3d indicate that pretreatment with PD173074-specific FGFR inhibitor or FMK, which binds in the C-terminal kinase domain of RSK and inhibits RSK autophosphorylation, nearly completely abolished formation of the FGFR2/RSK2 complex. On the other hand, application of BI-D1870, which binds to N-terminal kinase domain (NTKD) of RSK and inhibits RSK activity towards downstream substrates, while leaving the kinase itself phosphorylated, did not have any impact on FGFR2/RSK2 association. These data are in agreement with Nadratowska-Wesolowska et al., where BI-D1870 treatment had no effect on FGFR1-RSK2 complex formation either (FMK was not studied) [22]. Therefore, we conclude that formation of the FGFR2-RSK2 indirect complex upon FGF2 stimulation depends on FGFR2 and RSK2 activation rather than on RSK2 downstream signalling. Interestingly, in control conditions, we found that EGF treatment resulted in a slight increase in the amount of FGFR2-RSK2 complex (in agreement with previous report [22]), which may be due to the fact that EGF activates RSK2 through MAPK pathway, while FGFR2 remains unphosphorylated [18].

Intracellular trafficking of cell surface receptors modulates their signalling and cellular responses. Fibroblast growth factor receptors, like other receptor tyrosine kinases, in response to ligand binding undergo internalization from cell surface and controlled trafficking [22, 24, 25]. FGFR1 cleavage and internalization were proved to regulate pro-migratory gene expression. Inhibition of receptor trafficking abolished cell migration and invasive phenotype [24]. RSK2 was shown to bind directly to the C-terminal tail of FGFR1 and direct receptor endocytosis in human osteosarcoma cells [22]. We have found that stimulation with FGF2 promoted FGFR2 internalization; however pretreatment with specific RSK inhibitor (FMK) effectively prevented this effect. Subsequently, we examined a potential role of RSK2 in FGFR2 turnover in HB2 cells with stable RSK2 knockdown or treated with FMK. We found no difference in the level of FGFR2 expression or its ubiquitination resulting from RSK2 silencing or inhibition of RSK activity (Fig. 4b). This suggests that RSK2 might play a dual role for FGFR2 activities, i.e. it may mediate pro-migratory effects of FGFR2 in BCa cells [19] as well as be involved in regulation of FGFR2 intracellular trafficking.

To the best of our knowledge, this is the first study providing information on clinical significance of FGFR2/RSK2 interdependence in breast cancer patients. We demonstrated that FGFR2, RSK2 and RSK-P were expressed in breast cancer tissue and that patients with tumours devoid of RSK-P alone or in combination with FGFR2 had longer disease-free survival. Analysis of clinical material was supplemented by in vitro mechanistic analyses showing that FGFR2 and RSK2 formed an indirect complex in mammary epithelial cells. In addition, RSK activity was found to exert a significant impact on FGF2-triggered FGFR2 internalization. Taken together, our results imply that FGFR2-RSK2 signalling pathway is associated with BCa progression and evaluation of FGFR2/RSK-P expression might be useful in disease prognostication.

BCa, breast cancer; DFS, disease-free survival; EGF, epidermal growth factor; ER, oestrogen receptor; FGF2, fibroblast growth factor 2; FGFR1, fibroblast growth factor receptor 1; FGFR2, fibroblast growth factor receptor 2; FGFR3, fibroblast growth factor receptor 3; GSH, glutathione; HER2, human epidermal growth factor receptor 2; HR, hazard ratio; IGF-1, insulin-like growth factor 1; IHC, immunohistochemistry; IP, immunoprecipitation; PgR, progesterone receptor; RSK1, ribosomal s6 kinase 1; RSK2, ribosomal s6 kinase 2; RSK-P, phosphorylated ribosomal s6 kinases; RT-qPCR, reverse transcription quantitative polymerase chain reaction; SDS-PAGE, SDS polyacrylamide gel electrophoresis; SNP, single nucleotide polymorphism; TICs, tumour-initiating cells; TMA, tumour microarray; TNBC, triple-negative breast cancer.

## Electronic supplementary material

Table S1 (DOCX 21 kb)

Table S2 (DOCX 14 kb)

Figure S3 (GIF 22 kb)

High Resolution Image (TIFF 7179 kb)

Figure S4 (GIF 1 kb)

High Resolution Image (TIFF 566 kb)

Figure S5 (GIF 76 kb)

High Resolution Image (TIFF 8214 kb)
