# Supplementary material for: Interactions between FGFR2 and RSK2—implications for breast cancer prognosis
Source: Tumour Biol. 2016 Jul 30;37(10):13721–31. doi: 10.1007/s13277-016-5266-9 (PMC5097089; doi:10.1007/s13277-016-5266-9)
Supplement: Supplementary file 1 — (DOCX 21 kb) [file 13277_2016_5266_MOESM1_ESM.docx]

**Supplementary data. Table 1.**

|  | **FGFR2** | | | **RSK2** | | | **RSK-P** | | | **FGFR2/RSK-P** | | |
| --- | --- | --- | --- | --- | --- | --- | --- | --- | --- | --- | --- | --- |
|  |  |  |  |  |  |  |  |  |  | double- |  |  |
|  | negative | positive | p | negative | positive | p | negative | positive | p | negative | rest | p |
| **T stage** |  |  | 0.25749 |  |  | 0.68500 |  |  | 0.98599 |  |  | 0.49676 |
| stage 1-2 | 80 | 36 |  | 69 | 41 |  | 41 | 69 |  | 41 | 69 |  |
|  | 85.11% | 92.31% |  | 89.61% | 87.23% |  | 87.23% | 87.34% |  | 87.23% | 87.34% |  |
| stage 3-4 | 14 | 3 |  | 8 | 6 |  | 6 | 10 |  | 6 | 10 |  |
|  | 14.89% | 7.69% |  | 10.39% | 12.77% |  | 12.77% | 12.66% |  | 12.77% | 12.66% |  |
| **N stage** |  |  | 0.47067 |  |  | 0.81333 |  |  | 0.60366 |  |  | 0.27029 |
| stage 0 | 45 | 16 |  | 36 | 23 |  | 22 | 34 |  | 13 | 37 |  |
|  | 47.87% | 41.03% |  | 46.75% | 48.94% |  | 47.83% | 43.04% |  | 54.17% | 41.57% |  |
| stage 1-2 | 49 | 23 |  | 41 | 24 |  | 24 | 45 |  | 11 | 52 |  |
|  | 52.13% | 58.97% |  | 53.25% | 51.06% |  | 52.17% | 56.96% |  | 45.83% | 58.43% |  |
| **Grade** |  |  | 0.20023 |  |  | 0.62058 |  |  | 0.26168 |  |  | 0.65806 |
| grade 1-2 | 51 | 29 |  | 46 | 31 |  | 41 | 94 |  | 14 | 58 |  |
|  | 64.56% | 76.32% |  | 67.65% | 72.09% |  | 74.55% | 64.38% |  | 66.67% | 71.60% |  |
| grade 3 | 28 | 9 |  | 22 | 12 |  | 14 | 52 |  | 7 | 23 |  |
|  | 35.44% | 23.68% |  | 32.35% | 27.91% |  | 25.45% | 35.62% |  | 33.33% | 28.40% |  |
| **ER** |  |  | **0.00650** |  |  | 0.40451 |  |  | 0.59610 |  |  | 0.47315 |
| Negative | 47 | 9 |  | 32 | 16 |  | 17 | 33 |  | 11 | 34 |  |
|  | 49.47% | 23.68% |  | 41.56% | 34.04% |  | 36.96% | 41.77% |  | 45.83% | 37.78% |  |
| Positive | 48 | 29 |  | 45 | 31 |  | 29 | 46 |  | 13 | 56 |  |
|  | 50.53% | 76.32% |  | 58.44% | 65.96% |  | 63.04% | 58.23% |  | 54.17% | 62.22% |  |
| **PgR** |  |  | 0.27801 |  |  | 0.12529 |  |  | 0.10355 |  |  | 0.91880 |
| Negative | 37 | 11 |  | 30 | 12 |  | 12 | 32 |  | 8 | 31 |  |
|  | 38.95% | 28.95% |  | 38.96% | 25.53% |  | 26.09% | 40.51% |  | 33.33% | 34.44% |  |
| Positive | 58 | 27 |  | 47 | 35 |  | 34 | 47 |  | 16 | 59 |  |
|  | 61.05% | 71.05% |  | 61.04% | 74.47% |  | 73.91% | 59.49% |  | 66.67% | 65.56% |  |
| **HER2** |  |  | 0.24586 |  |  | 0.92326 |  |  | 0.73210 |  |  | 0.41236 |
| Negative | 65 | 30 |  | 56 | 33 |  | 32 | 55 |  | 15 | 64 |  |
|  | 82.28% | 90.91% |  | 87.50% | 86.84% |  | 82.05% | 84.62% |  | 78.95% | 86.49% |  |
| Positive | 14 | 3 |  | 8 | 5 |  | 7 | 10 |  | 4 | 10 |  |
|  | 17.72% | 9.09% |  | 12.50% | 13.16% |  | 17.95% | 15.38% |  | 21.05% | 13.51% |  |
| **Molecular**  **subtype** |  |  | 0.07813 |  |  | 0.74463 |  |  | 0.93414 |  |  | 0.34639 |
| HR+, HER2- | 48 | 27 |  | 44 | 29 |  | 27 | 42 |  | 10 | 54 |  |
|  | 60.00% | 84.38% |  | 68.75% | 76.32% |  | 69.23% | 65.63% |  | 52.63% | 72.97% |  |
| HR+, HER2+ | 6 | 2 |  | 3 | 2 |  | 3 | 4 |  | 2 | 5 |  |
|  | 7.50% | 6.25% |  | 4.69% | 5.26% |  | 7.69% | 6.25% |  | 10.53% | 6.76% |  |
| HR-, HER2+ | 10 | 1 |  | 5 | 3 |  | 4 | 7 |  | 2 | 6 |  |
|  | 12.50% | 3.13% |  | 7.81% | 7.89% |  | 10.26% | 10.94% |  | 10.53% | 8.11% |  |
| TNBC | 16 | 2 |  | 12 | 4 |  | 5 | 11 |  | 5 | 9 |  |
|  | 20.00% | 6.25% |  | 18.75% | 10.53% |  | 12.82% | 17.19% |  | 26.32% | 12.16% |  |
| **Histological**  **type** |  |  | 0.12192 |  |  | 0.11157 |  |  | 0.16642 |  |  | 0.98400 |
| Ductal | 75 | 27 |  | 62 | 31 |  | 32 | 65 |  | 18 | 71 |  |
|  | 88.24% | 77.14% |  | 87.32% | 75.61% |  | 78.05% | 87.84% |  | 85.71% | 85.54% |  |
| Lobular | 10 | 8 |  | 9 | 10 |  | 9 | 9 |  | 3 | 12 |  |
|  | 11.76% | 22.86% |  | 12.68% | 24.39% |  | 21.95% | 12.16% |  | 14.29% | 14.46% |  |
