# Supplementary material for: Interactions between FGFR2 and RSK2—implications for breast cancer prognosis
Source: Tumour Biol. 2016 Jul 30;37(10):13721–31. doi: 10.1007/s13277-016-5266-9 (PMC5097089; doi:10.1007/s13277-016-5266-9)
Supplement: Supplementary file 2 — (DOCX 14 kb) [file 13277_2016_5266_MOESM2_ESM.docx]

**Supplementary data. Table 2.**

|  | **N** | ***FGFR2* average gene expression (±SD)** | **p** | **N** | ***RSK2* average gene expression (±SD)** | **p** |
| --- | --- | --- | --- | --- | --- | --- |
| **T stage** |  |  |  |  |  |  |
| stage 1-2 | 88 | 1,78 (0,71) | 0.35 | 88 | 0,78 (0,32) | 0.51 |
| stage 3-4 | 9 | 1,96 (0,80) |  | 9 | 0,80 (0,18) |  |
| **N stage** |  |  |  |  |  |  |
| stage 0 | 60 | 1,85 (0,73) | 0.52 | 60 | 0,77 (0,33) | 0.56 |
| stage 1-2 | 36 | 1,76 (0,65) |  | 36 | 0,77 (0,29) |  |
| **Grade** |  |  |  |  |  |  |
| grade 1-2 | 51 | 1,79 (0,64) | 0.81 | 51 | 0,68 (0,27) | **0.02** |
| grade 3 | 30 | 1,81 (0,92) |  | 30 | 0,88 (0,35) |  |
| **ER** |  |  |  |  |  |  |
| negative | 44 | 1,82 (0,72) | 0.54 | 44 | 0,79 (0,36) | 0.62 |
| postive | 51 | 1,87 (0,65) |  | 51 | 0,76 (0,26) |  |
| **PgR** |  |  |  |  |  |  |
| negative | 40 | 1,77 (0,83) | 0.28 | 40 | 0,87 (0,31) | **0.02** |
| Positive | 55 | 1,87 (0,55) |  | 55 | 0,69 (0,29) |  |
| **HER2 (FISH)** |  |  |  |  |  |  |
| Negative | 72 | 1,83 (0,74) | 0.94 | 72 | 0,78 (0,34) | 0.35 |
| Positive | 14 | 1,77 (0,58) |  | 14 | 0,72 (0,16) |  |
| **Histological type** |  |  |  |  |  |  |
| Ductal | 79 | 1,75 (0,75) | 0.38 | 79 | 0,75 (0,31) | 0.39 |
| Lobular | 7 | 2,01 (0,45) |  | 7 | 0,78 (0,36) |  |
