# Supplementary figures and images for: Interactions between FGFR2 and RSK2—implications for breast cancer prognosis
Source: Tumour Biol. 2016 Jul 30;37(10):13721–31. doi: 10.1007/s13277-016-5266-9 (PMC5097089; doi:10.1007/s13277-016-5266-9)

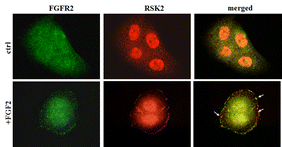

Supplement: Supplementary file 3 — (GIF 22 kb) [file 13277_2016_5266_Fig5_ESM.gif]

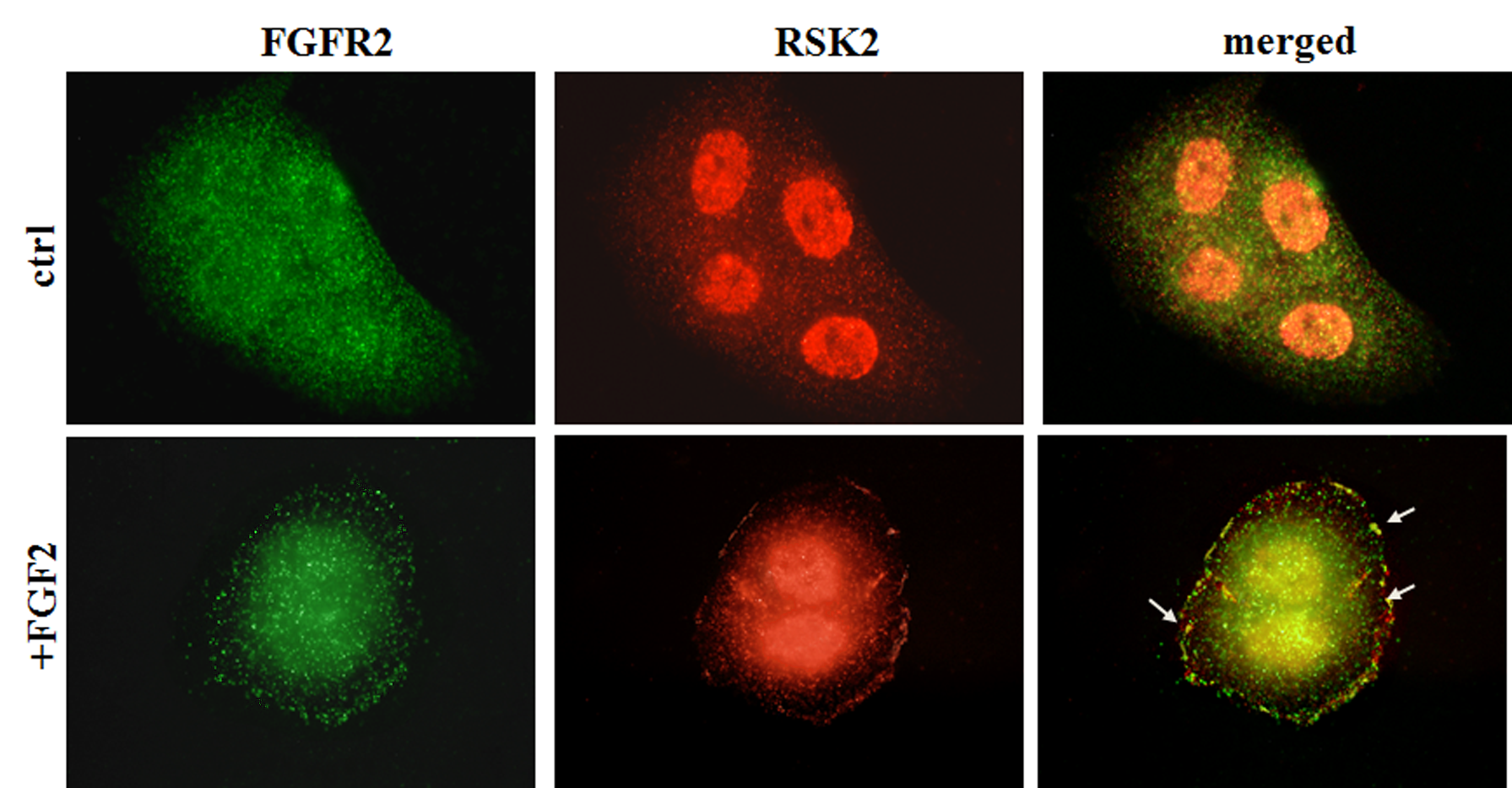

Supplement: Supplementary file 4 — High Resolution Image (TIFF 7179 kb) [file 13277_2016_5266_MOESM3_ESM.tif]

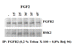

Supplement: Supplementary file 5 — (GIF 1 kb) [file 13277_2016_5266_Fig6_ESM.gif]

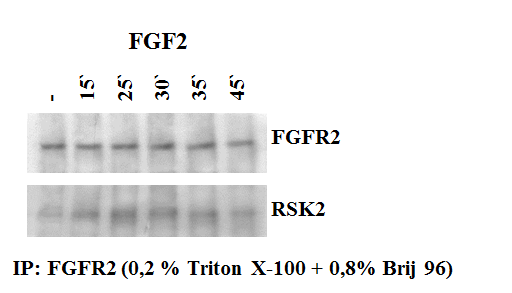

Supplement: Supplementary file 6 — High Resolution Image (TIFF 566 kb) [file 13277_2016_5266_MOESM4_ESM.tif]

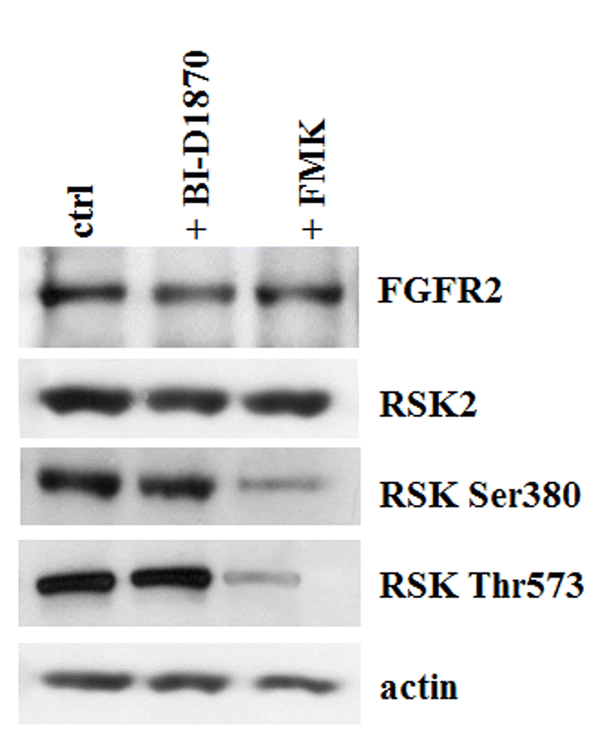

Supplement: Supplementary file 7 — (GIF 76 kb) [file 13277_2016_5266_Fig7_ESM.gif]

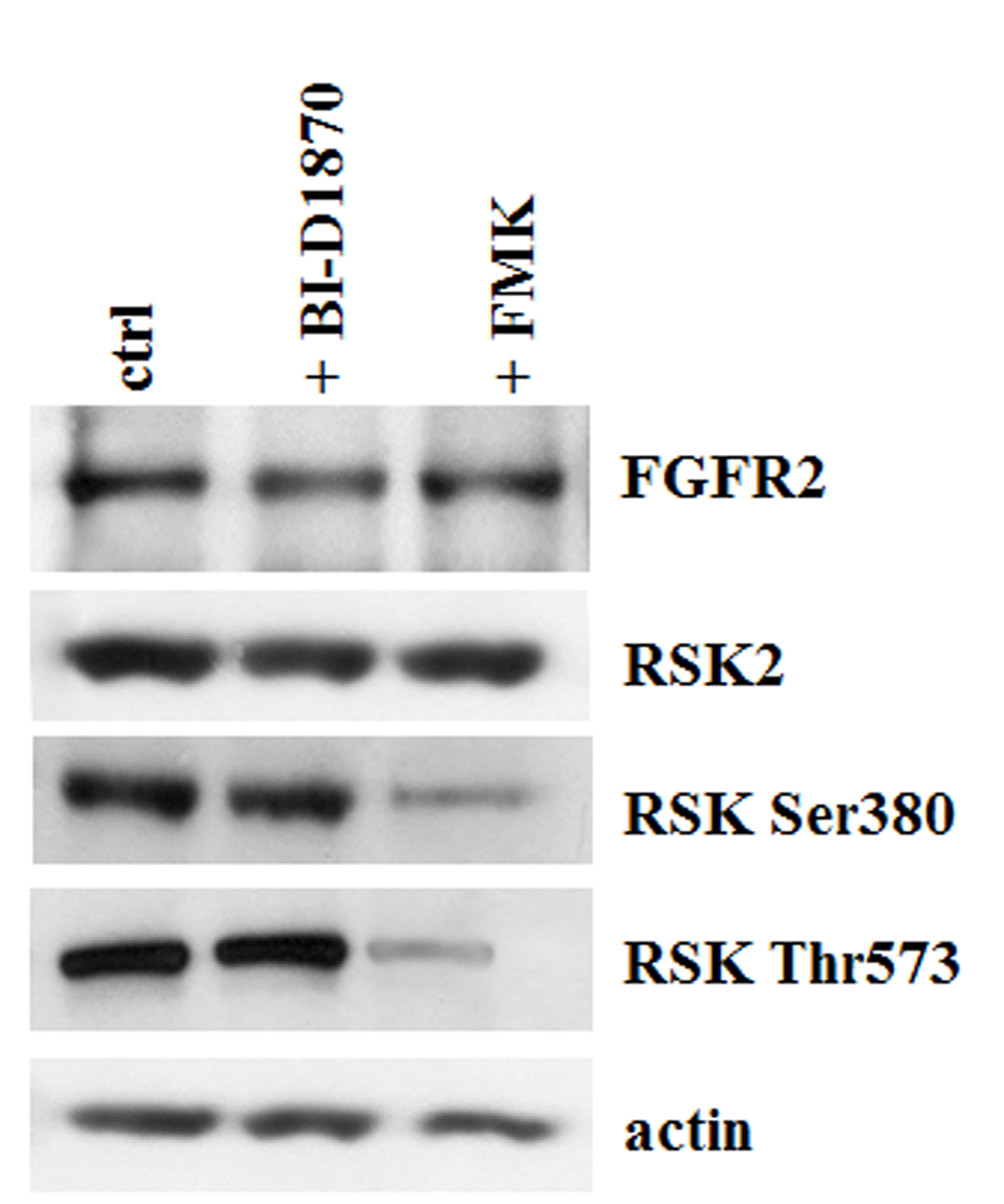

Supplement: Supplementary file 8 — High Resolution Image (TIFF 8214 kb) [file 13277_2016_5266_MOESM5_ESM.tif]
